# Influence of Industrial Metakaolin Waste on Autoclaved Fiber Cement Properties Changes in Standard Fire Environment

**DOI:** 10.3390/ma15103455

**Published:** 2022-05-11

**Authors:** Tomas Veliseicik, Ramune Zurauskiene, Modestas Kligys, Mark Dauksevic

**Affiliations:** 1 Faculty of Civil Engineering, Vilnius Gediminas Technical University, 10223 Vilnius, Lithuania; ramune.zurauskiene@vilniustech.lt (R.Z.); modestas.kligys@vilniustech.lt (M.K.); 2 Expertise Division, Fire Research Centre, 13221 Valčiūnai, Lithuania; mark.dauksevic@vpgt.lt

**Keywords:** metakaolin, tobermorite, fiber cement, standard fire curve

## Abstract

An investigation was conducted on the influence that industrial metakaolin waste (IMW) has on the properties of autoclaved fiber cement composition (FCC) samples. FCC samples were made from fiber cement plate’s typical components using the same proportions. In samples, IMW was used instead of cement in 10%, 20%, 30% proportions and in 50%, 100% proportions instead of ground quartz. Differential thermal analysis (DTG), thermogravimetric analysis (TGA), ultrasound pulse velocity (UPV), density, porosity and optical microscope (OM) research methods were used to identify the micro and macrostructure of samples. Mechanical properties were evaluated using flexural and compressive strength research methods. It was established that IMW was used instead of cement in fiber cement composition samples up to 10% and in fiber cement composition samples instead of ground quartz forms density microstructure structure because of Al-rich tobermorite. As a result, the flexural and compressive strength increased. Samples with higher content of IMW instead of cement had unreacted IMW and a less dense microstructure. In this case, flexural and compressive strength decreased. All FCC samples were fired in a standard fire curve (ISO 842) for 30 min. Samples of mechanical properties were established by doing flexural and compressive strength tests, and which results showed the same trends.

## 1. Introduction

Fiber cement belongs to the cement matrix composite group. Due to its good technical properties, fiber cement is widely used in the building industry. Fiber cement plates are used in interior wall coverings, balcony and terrace enclosure installation, chimney covering, roof hanging, etc. [1]. However, the main fiber cement plate usage is for installations of ventilation façade systems during new building constructions and renovations. Events during the last few years have shown that it is important for ventilation façade materials properties to show reactions to fire and high temperatures.

After analyzing publicly available statistics on fire rises, it was determined that 35.5% of fires start in buildings [2]. Lately, due to the heightened use of synthetic polymer material in furniture, household appliances and decoration production, fires now more commonly spread from the building to the exterior. Several scientists have done experimental research, natural tests, mathematical models and examinations of flame and fire spreading mechanisms between building floors [3,4,5,6], and they state that façade system flammability and resistance to fire assurance are priority technical indicators. To ensure these indicators, façade systems have to withstand high-temperature effects for a certain amount of time.

It has been known for a long time that, in the general case, concrete, that has Portland cement in its composition, cracks and splits at a high temperature. It was examined that, when affected by high temperatures, complex physicochemical and physicomechanical processes take place in concrete that determine its destruction. Experimental research showed that a high-temperature destructive effect on concrete is most often related to warmth, tension and humidity effects.

Ventilated façade with fiber cement plates fire experiments have shown that these products split within 10 min from the start of the experiment, that is when the temperature reaches about 250 °C. In certain cases, the splitting of fiber cement plates was followed by explosion noises and separated plate fragments [7]. Fiber cement plate resistance in times of a fire when high temperatures were reached is examined in this article [8]. It was determined that the dehydration process starts from 100 °C and is affected by chemical reactions as well as polymorphic structural changes, which become more significant for final product properties as temperature rises. To raise the resistance to high temperatures, it is necessary to modify the composition of the existing product and use components resistant to high temperatures and their combinations. This component usage can determine a more stable to high temperatures component formation during the hydration and/or hardening processes, as well as compositions, which, after splitting, cause a lower amount of autoclave fiber cement product destruction during dehydration. When looking into this, concrete’s resistance to high temperatures can be raised by changing out binders and aggregate and by using additives and admixtures [1].

Pozzolanic additives may be used for the listed goals. Pozzolans are composed of silicate, aluminosilicate, or aluminate materials, which tend to have weak cementing properties (or do not have these properties at all). In environmental temperatures, when there is humidity, these materials chemically react with calcium hydroxide, creating compounds that tend to have cementing properties and improve cement-based system strength and durability [2]. Up to now, there have been quite a few researches in which pozzolanic additives are used for raising cement material resistance to fire. Lublóy [9] researched pozzolanic additive, crushed fly ash, effect on cement material properties in high temperatures. Carried out research showed that concrete sample thermal destruction happened at around 400 °C temperature. Visual observation of the samples showed thermal cracks, while samples that had crushed fly ash and quartz sand aggregate did not have any thermal, visual cracks at 800 °C temperature [3].

A lot of research is carried out using silicate microdust as a pozzolanic additive for concrete constructions and evaluating their properties at high temperatures [4,5,6]. Saad [10] studied concrete samples with 10%, 20% and 30% silicate microdust (SiO_2_), samples being heated up to 600 °C. Sample results showed that the best result was reached when concrete samples had 10% silicate microdust (SiO_2_). Samples with 20% and 30% silicate microdust (SiO_2_) had worse results; however, even their compressive strength was higher by 28% than the control sample without additives.

Metakaolin (MK) is anhydrous aluminosilicate material produced from thermally heated kaolin. The main part of kaolin’s composition is kaolinite—made by heating it between 600 and 800 °C temperature and dehydrating it. Due to heating, the kaolinite hexagonal layered structure is practically decomposed, and its polymorphic change into an amorphous phase takes place [11]. Thermal reaction time and temperature directly affect MK chemical composition and its activity. Kaolinite thermal cracking chemical reaction is described as:

Al_2_O_3_·2SiO_2_·2H_2_O → Al_2_O_3_·2SiO_2_ + 2H_2_O (1)

Due to the unstable Al-O atom layout, MK is more reactive in an alkaline environment than kaolin is [12].

Researchers that studied MK pozzolanic effect on cement materials and their properties determined their positive effect on final product properties [13,14]. MK can also be successfully used in really strong concrete production. Its optimal amount (10%) ensures the best sample properties [15]. MK’s effect on samples at 800 °C was determined by researchers [16]. Research results showed that strong concrete samples remaining compression strength up to 400 °C temperature was higher in samples that used MK. Up to this temperature treatment, the highest remaining compression strength was in samples with 20% MK, and strength proportionally lowered as the MK amount lowered. The 600 °C and higher temperature strength concrete samples with MK remaining compression strength were lower than the controlled sample results. Regular concrete samples with MK additive remaining compression strength result tendencies in higher temperatures were similar when compared with strong concrete samples. Near 600 °C temperature remaining compression strength sample with MK additive was similar to the controlled sample, while near 800 °C temperature, in some cases, was slightly smaller.

Ilić [17] researched autoclave material properties using mechanically active kaolin additives. Research results showed that during the autoclave hardening process, active kaolin additives raise compression strength due to Ca(OH)_2_ (CH) lowering and crystal tobermorite formation. Matsui [18] carried out in-site research with autoclave porous concrete samples by addition using silica and Al additives. Results showed that silicate additives in hydrothermal conditions formed tobermorite while Al-additives had an influence on Al tobermorite formation.

It can be concluded that MK determines the properties of cement materials due to the three main actions. That is filler effect, improvement of regular Portland cement hydration process, and pozzolanic reaction with Ca(OH)_2_ (CH) [16].

After literature analysis, it was determined that the MK usually used was of uncomplicated systems, which are composed of three to four components for the experiments. Currently, there is no investigated MK additive effect on autoclave fiber cement materials, which have a complicated composition (closer to production) when those materials enter into high temperatures. The dehydration process and macro and microstructure behavior of these component combustions are not clear. Further, it is not clear what kind of effect MK has on these autoclave fiber cement material mechanical properties. This work attempts to investigate all these processes.

## 2. Materials and Methods

**Figure 1:**
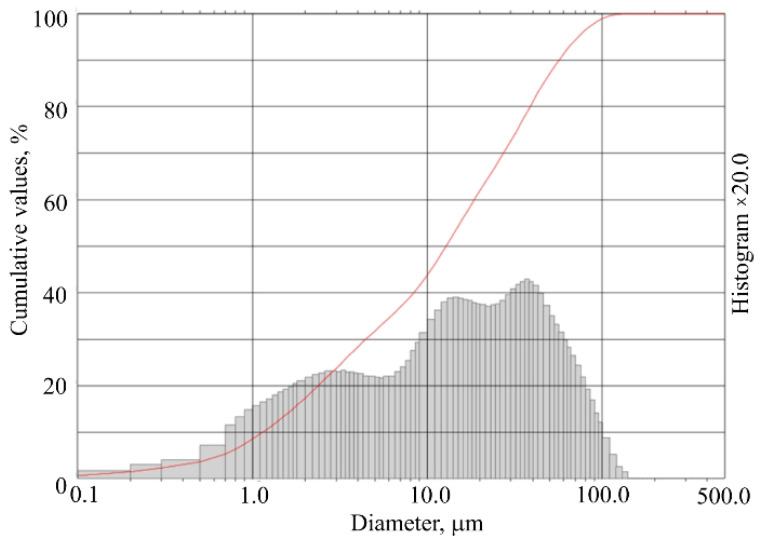
Distribution of Portland cement particles by size.

Portland cement CEM I 42.5 N, produced by a Lithuanian company and complying with EN 197-1 requirements, was used as binding material. The mineralogical composition of clinker (Bogue calculation): C_3_S—68.1%; C_2_S—13.8%, C_3_A—8.6%; C_4_AF—7.1% [19]. The chemical composition of Portland cement and the distribution of its particles by size are presented in Table 1 and Figure 1.

**Figure 2:**
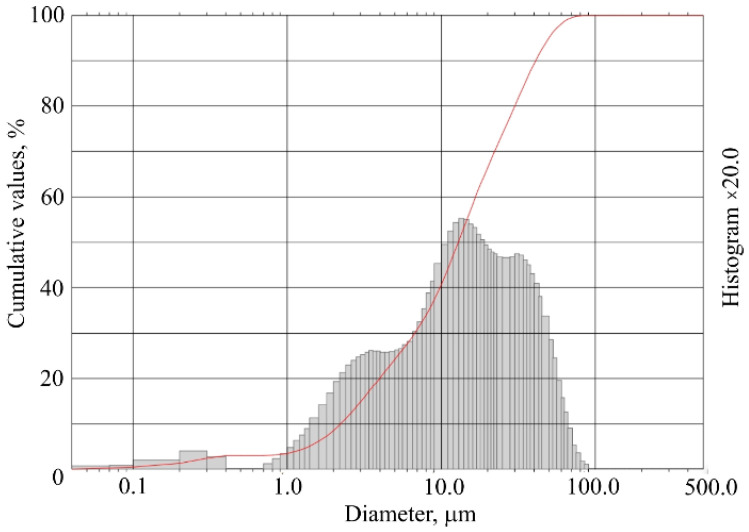
Distribution of milled quartz sand particles by size.

Quartz sand (M32 and M300) is composed of 99.5% silicate dioxide and 0.5% other admixtures (Al_2_O_3_, Fe_2_O_3_, TiO_2_, K_2_O, CaO). Quartz sand M300 from JSC “Anyksčių kvarcas”, Anyksciai, Lithuania is milled in a circular mill until it reaches a specific surface of 4400 cm^2^/g. The distribution of milled quartz sand particles by size is presented in Figure 2.

**Figure 3:**
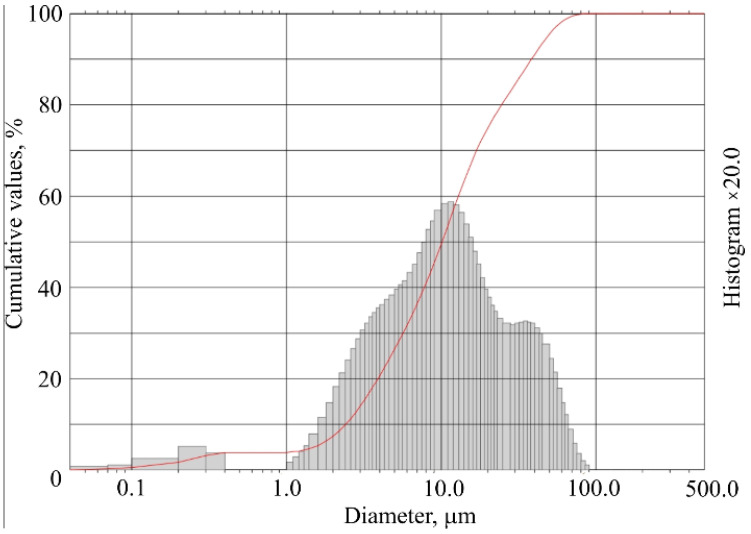
Distribution of MKA particles by size.

MKA is a waste generated in Lithuania from the production of foamed glass granules. The semi-finished foam glass granulation is pulverized with kaolin and placed in a furnace at 800–820 °C temperature. The function of kaolin is to protect the already swelling granules from sticking to the walls and surface of the furnace and to each other. Metakaolin in production is used as a material for separating granules as it does not react with granules at 800–820 °C temperature. MKA is produced when sieving produced foam glass granules. The chemical composition of MKA and the distribution of MKA particles by size are presented in Table 1 and Figure 3.

**Figure 4:**
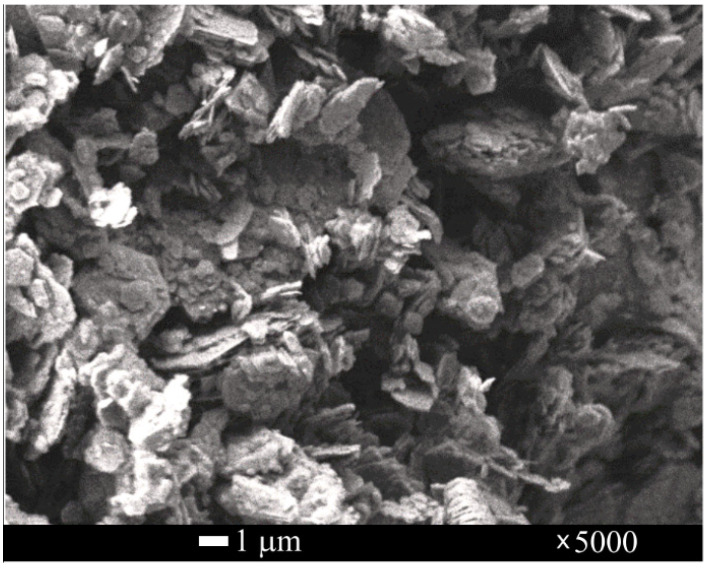
SEM image of the MKA zoomed in 5000 times.

A microstructure image of MKA was obtained with the field emission scanning electron microscope (SEM) JSM-7600F (accelerating voltage—4.0 kV) (JEOL, Tokyo, Japan). An opportunity to research an uncovered electrically conductive layer surface and to perform an investigation of changes appearing on the chosen surface place due to the heating arises. Figure 4 shows that MKA consists of irregular shape plates.

An armored fiber made up of two types of cellulose was used. The first type of cellulose fibers was shorter (5.45%), and other types of cellulose fibers (1.64%) formed a so-called “hand fan”.

The research was carried out by mixing a combination of raw materials that were close to the mixture used for the industrial production of façade fiber cement plates (Table 2, mixture marking E). Portland cement or milled quartz sand were partially replaced by the metakaolin waste (MKA) in regular fiber cement plate mixture (Table 2, mixture markings C and MKS). This was done with the intention that MKA additive improves the behavior of autoclave fiber cement material in higher temperatures as well as its properties after high temperatures. Table 1 shows tried mixtures and sample components made from them. There were 10%, 20% and 30% of Portland cement replaced (by mass) with MKA in the mixtures, marked as C10, C20 and C30. Further, 50% and 100% of milled quartz sand (M300) were replaced (by mass) with MKA in the mixtures, marked as MKS50 and MKS100. The lowest amount of Portland cement was used in the mixture, marked as C30 (composed only of 27% of Portland cement). In the other mixtures, the amount of Portland cement was higher. A mixture, marked as MKS100, was mixed without using the milled quartz sand.

Table 2 shows the amount of dry raw materials used in the different mixtures. Tap water (temperature 20 ± 2 °C) was used for the preparation of these mixtures. The water to cement ratio in the mixtures was 0.3. Milled sand and cellulose fiber were prepared in a factory that produces autoclaved fiber cement plates. Cellulose fiber was factory separated in water (stored in the closed buckets). The amount of water in the cellulose fiber was determined, and the total amount of water in the mixture was adjusted before the mixing.

Mixtures were mixed in the laboratory mixer (Hobart type) (BRIO Hranice, Hranice, Czech Republic) with a forced blade movement, mixing mass at the speed of 45 rpm for 5 min. This process was carried out similarly to the industrial production of facade fiber cement plates. The prepared mixtures were poured into the metal prism-shaped forms (sized 40 mm × 40 mm × 160 mm) and compacted on a vibrating table, which is used for mortars. 0.5 kg load was used on the top of the samples during the compaction. Samples were kept in the metal forms for 8 h, and afterward, the samples with the forms were put into the autoclave. Samples were hardened in the autoclave for 10.5 h (pressure—10.13 bar, temperature—179 °C). The rising of the pressure in the autoclave was performed for 2 h, the samples were held at the highest pressure for 7 h, and the pressure was released for 1.5 h. The samples were held in an environment of 60% humidity for 14 days after hardening in the autoclave.

The investigations of the macrostructure of samples were carried out by an optical microscope “Motic” (Motic, Xiamen, China) with a digital camera “Pixera PVC 100C” (Pixera Corporation, Santa Clara, CA, USA), which was connected to a computer.

Samples for the dry density determination were dried in a laboratory climatic chamber at 100 °C ± 5 °C temperature until constant mass. Samples were conditioned according to the requirements of standard EN 13238 in a conditioning room at 22 °C ± 2 °C temperature and 50% ± 5% humidity when determining sample air-dry density.

Sample absorption kinetics were determined by soaking them in water at +20 °C ± 2 °C temperature. Samples were dried in a laboratory climatic chamber at 100 °C ± 5 °C temperature until constant mass before the experiment. Water absorption in the starting period was determined to be 0.5 h, 1 h, 3 h, 6 h, 12 h, 24 h and after weighting the samples, it was repeated for each day until the mass change of the sample was smaller than 0.1%. Before every weighting, the leftover water was wiped away from the sample. Sample absorption kinetics was determined in percentages using this formula:

(2) $$W=\frac{m_{1}-m_{0}}{m_{0}}\times 100,\;\%$$

where:

W—water absorption, %;

*m*_0_—sample mass after climatic chamber drying, g;

*m*_1_—soaked sample mass, g.

The development of structure in the samples was evaluated by means of the ultrasonic pulse velocity method, using the tester “Pundit 7” (CNS Farnell, Hertfordshire, UK). The values of ultrasonic pulse velocity were obtained after hardening and drying the samples. Samples were placed between two ultrasonic transducers (transmitter and receiver) operating at a frequency of 54 kHz. The transducers were pressed against the samples at two strictly opposite points. Vaseline was used to ensure good contact. The ultrasonic pulse velocity was calculated using this equation:

(3) $$V=\frac{l}{t}\times 10^{6}$$

where:

*l*—distance between cylindrical heads, m;

*t*—time of pulse spread, s. The compressive strength test was conducted in the powerful electromechanical testing machine H200kU of capacity 200 kN and load measurement accuracy ±0.5% of applied load, from 0.2% to 100% capacity. The speed of load increment was 60.0 N/s until the destruction of the sample. The concluding test result was taken as the average value calculated out of at least five successful measurements. The compressive strength of the sample was received from the following Equation (4):

(4) $$f_{c}=\frac{F}{A_{c}}$$

where:

*F*—maximal failure load, N;

*A_c_*—cross-section area of the tested sample, mm^2^.

Flexural strength testing equipment was used. At length-half of each sample, the linear load was applied across the width (3 bending points). The speed of increment of bending load was 14 N/s. The final test result was taken average value calculated out at least three successful measurements. The flexural strength of the sample was calculated from the following equation:

(5) $$f_{cf}=\frac{1.5Fl}{bh^{2}}$$

where:

*F*—maximal applied bending load, N;

*l*—span between the supports, mm;

*b*—width of the tested sample, mm;

*h*—height of the tested sample, mm.

**Figure 5:**
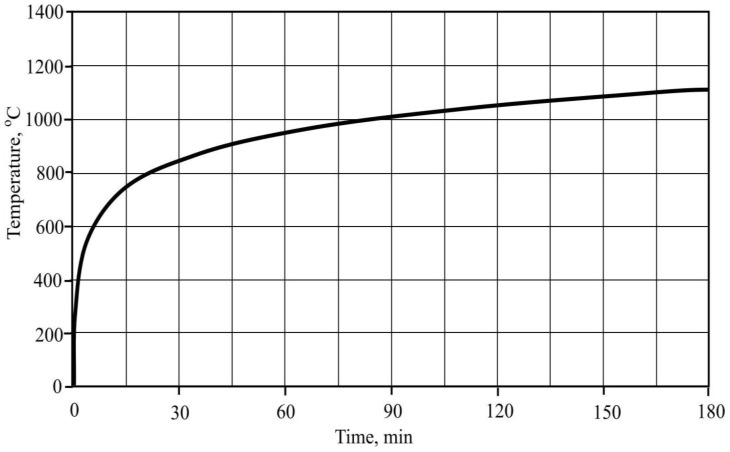
Standard fire curve.

The sample temperature was affected using a heating chamber according to the standard fire curve (ISO 834), which describes Equation (6), the graphic expression shown in Figure 5. Samples were added into the heating environment in groups of 3 while making sure each side of the sample surface would be affected by the temperature. After 30 min, the sample temperature reached 842 °C. The experiment was stopped, and the samples were cooled down in a natural way. After cooling them to the laboratory room temperature, samples were carried over to the conditioning room with 22 °C ± 2 °C temperature and 50% ± 5% humidity.

(6) $$\theta_{g}=20+345\log_{10}\left(8t+1\right)$$

where:

*θ_g_*—fire temperature, °C;

*t*—time, min.

Differential thermogravimetric analysis (DTG) and thermogravimetric analysis (TG) were carried out using the thermogravimetric analyzer “Linseis STA 1600” (Selb). During the experiment, the temperature was measured using K type thermocouple using an aluminum oxide (Al_2_O_3_) crucible with a cover. Thermal analysis was done in a nitrogen gas environment, ensuring the nitrogen flow was 0.04 L/min. Experiments were carried out from 35 °C to 1000 °C isotherms. For this heating regime, a 10 °C/min temperature rising speed was determined. The samples were milled in a circular mill Pulverisette 6 (FRITSCH). The sample weight swung between 78 and 83 mg. Results were shown in graphs, where the abscissa axis shows according to the temperature curve, while the ordinate shows thermogravimetry and differential thermogravimetry results.

## 3. Results

The mass for fiber cement plate production is composed of binding materials—Portland cement, aggregates and armored cellulose fibers. Hardened Portland cement is a matrix that holds bonded aggregates—sand and armoring materials—cellulose fibers. This hardening process is very difficult; during it, hydration happens at different speeds on various sizes, forms and property cement components. Further, in this system, additives have a role: aluminum hydroxide and kaolin, while the hardening happens in autoclave conditions.

**Figure 6:**
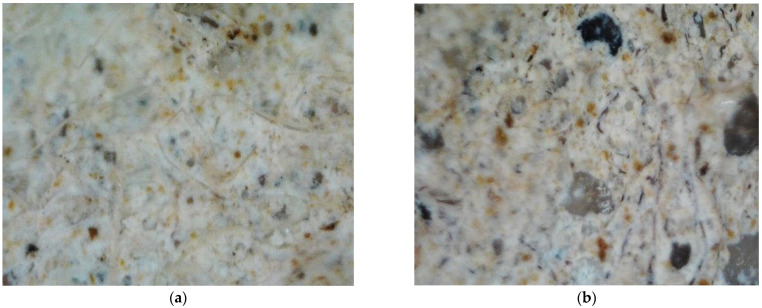
Sample E macrostructure zoomed in 12 times: (**a**) before fire effect; (**b**) after fire effect.

**Figure 7:**
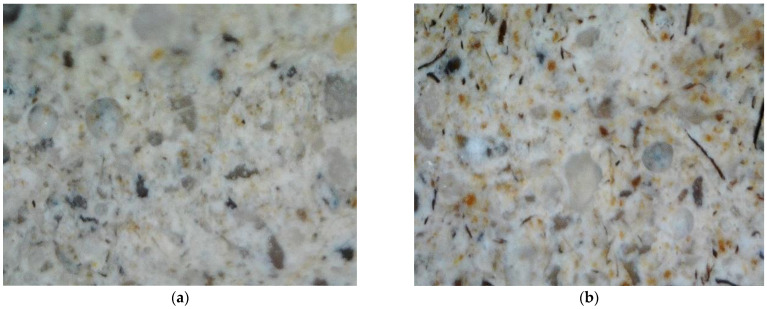
Sample C30 macrostructure zoomed in 12 times: (**a**) before fire effect; (**b**) after fire effect. Cement, MKA and milled quartz particle size determination research showed that used MKA average particle size is 15.34 μm, milled quartz sand is 17.66 μm, while cement is 21.71 μm.

Sample surface research carried out using an optic microscope showed macrostructure formation until MKA usage (Figure 6) and after MKA usage (Figure 7). The sample after fire effect macrostructure research showed burnt-out cellulose fiber and specific pore formation (Figure 6b and Figure 7b).

After carrying out the research, it was determined that samples formed using MKA particles are 15.13% smaller than milled quartz sand and 41.53% smaller than cement particles.

**Figure 8:**
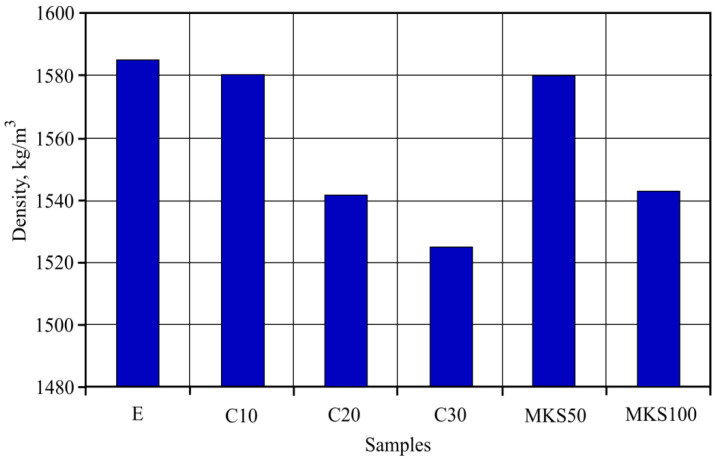
Researched sample dry density results.

After hardening, dry sample density was determined. Dry density results are shown in Figure 8.

It can be seen that by adding MKA instead of cement, the sample density lowered. Density lowered by a small bit, only up to 4% (C30 samples). When adding MKA into the formation mass, instead of quartz sand, the sample density also lowered by 3% (MKS100 samples). It can be seen that sample density rose or that the lowering was not very noteworthy, swinging between 5% boundaries. A more noteworthy dry density change is seen in MKS100 and C20 samples.

**Figure 9:**
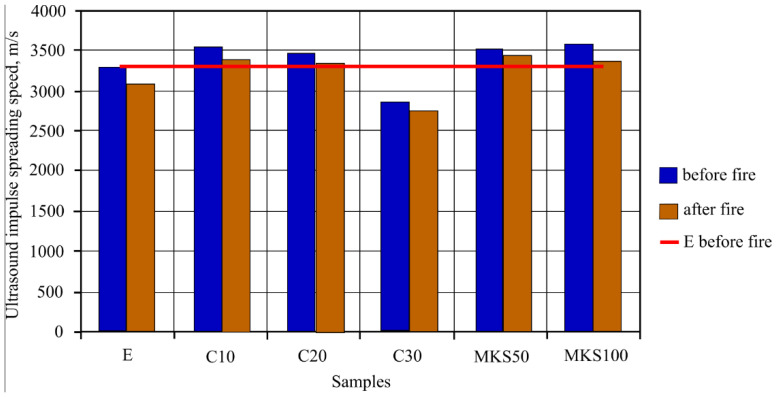
Sample ultrasound impulse spreading speed research results.

Ultrasound impulse splitting speed research was carried out, the results of which are shown in Figure 9. In samples C10 and C20, it was determined that ultrasound splitting speed was greater than E, while the C30 sample was lower by even 12.96%. MKS50 sample ultrasound splitting speed was the highest of all studied samples and 8.91% bigger than E. The MKS100 sample ultrasound splitting sound was slightly lower than MKS50 and C10; however, it was higher than E. After the fire effect was carried out in sample ultrasound impulse spreading speed research, result tendencies were similar to samples before the fire effect. C10 and C20 samples had ultrasound spreading speed that was higher than sample E, while the C30 sample speed was lower by 10.63%. In this case, the MKS50 sample determined ultrasound spreading speed was the highest out of all samples and higher than sample E by 11.82%. MKS100 sample ultrasound spreading speed was lower than MKS50 and C10; however, it was still higher than sample E.

**Figure 10:**
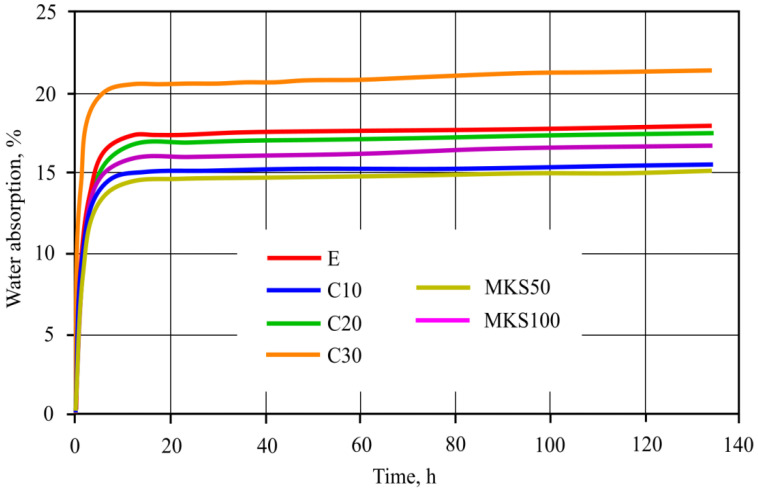
Sample water absorption results.

Sample absorption kinetics were determined and shown in Figure 10. Research results showed that C30 samples had the highest absorption, which after 134 h reached 21.35%. The smallest absorption was of MKS50 samples and was equal to 15.12%. All sample absorption, except for C30, when compared to E samples, was smaller.

**Figure 11:**
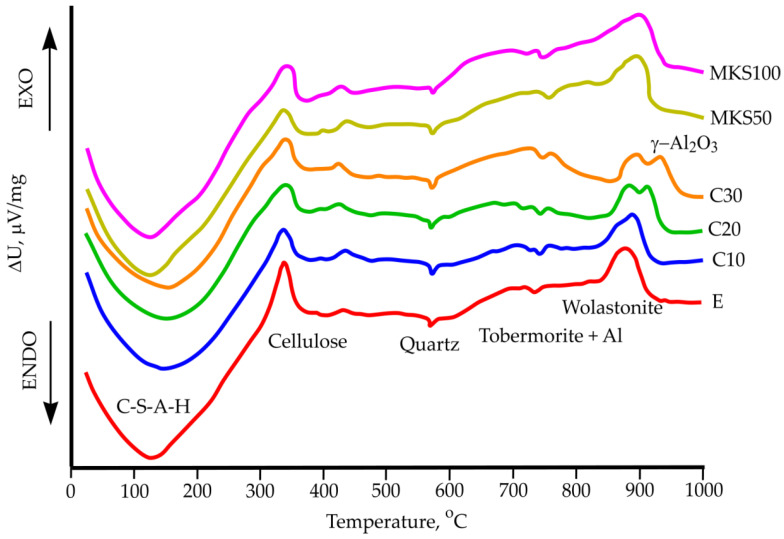
Sample DTG results.

**Figure 12:**
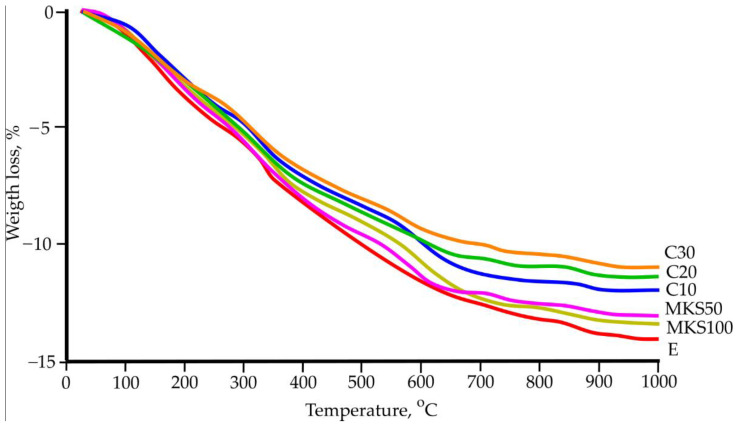
Sample TGA results.

DTG and TGA research was carried out (Figure 11 and Figure 12). After carrying out multiple types of research with the same samples, research results were analogous. General mass loss at 1000 °C temperature sways between 14.13% and 11%. Several exothermic effects were determined, which occurred at 350 °C, 420 °C, 880 °C, and 920–930 °C temperatures and two endothermic effects occurred at 130–150 °C and 571 °C temperature.

**Figure 13:**
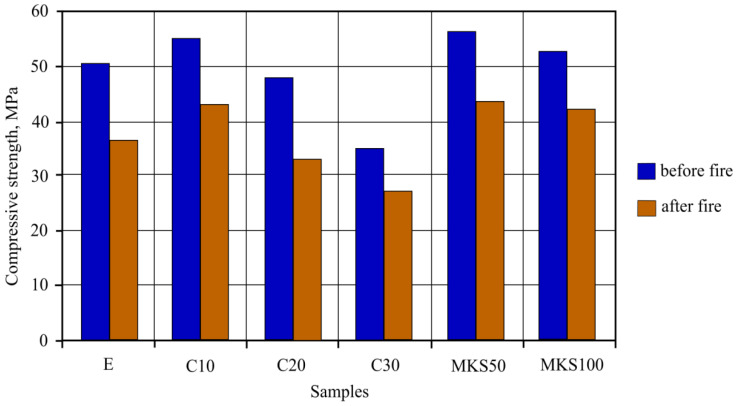
Sample compressive strength before and after fire effect results.

Compression strength research results showed that the C10, MKS50 and MKS100 sample compression strength was higher than E samples (Figure 13). The highest compression strength was determined in MKS50 samples, and it was 11.1% higher than in E samples. Similar results were obtained in both C10 and MKS100 samples. The determined compression strength in these samples was accordingly 9.1% and 4.3% higher than in E samples. C20 sample compression strength was lower by 4.9%. Noticeable sample compression strength lowering was observed in C30 samples, which reached 31.2%.

After the 30 min standard fire effect, compression strength research was carried out using experiment samples. Sample compression strength from starting strength lowered from 30.9% to 20.4%. Samples C10, MKS50 and MKS100 compression strength lowered in a similar range and are approximately about 5% when compared to E sample results.

**Figure 14:**
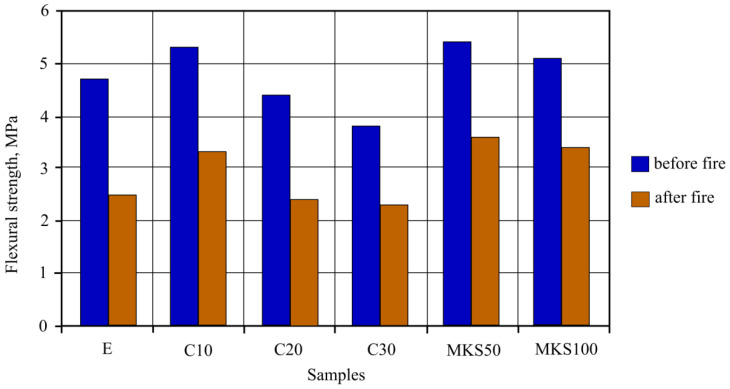
Sample bending strength before and after fire effect results.

Bending strength sample results (Figure 14), as well as compression strength sample results, showed that C10, MKS50 and MKS100 sample bending strength was higher than E samples. The highest bending strength was determined in MKS50 samples as well, and it was 14.9% higher than in E samples. The C10 and MKS100 samples determined bending strength was accordingly 12.8% and 8.5% higher. The C20 sample bending strength was lower by 6.3%. The highest bending strength decrease was determined in C30 samples, which reached 19.1%.

After the 30 min standard fire effect, bending strength research was carried out using experiment samples. Sample bending strength from starting lowered from 45.5% to 33.3%. Samples MKS50 and MKS100 bending strength lowering is the same, and they are smaller than 13.5% when compared to E samples. C10 sample bending strength lowering was smaller than in E samples by 9.1%.

## 4. Discussion

From references, it is known that MKA takes place in the cement hydration process by forming tobermorite. Firstly, cement minerals alite and belite hydrate forming cement hydration products according to the following Equations (7) and (8):

2(3CaO·SiO_2_) + 11H_2_O → 3CaO·SiO_2_·8H_2_O + 3(CaO·H_2_O) + *heat* (7)

2(2CaO·SiO_2_) + 9 H_2_O → 3CaO·SiO_2_·8H_2_O + CaO·H_2_O + *heat* (8)

Main hydration products are calcium hydrosilicates (CSH) and portlandite (CH). The latter reacts with MKA forming calcium aluminum hydrosilicates (CSAH) and tobermorite, usually, 11A, while chemical reaction Equations (9)–(11) depend on MKA and portlandite (CH) ratio [22]:

Al_2_O_3_·2SiO_2_+ 6(CaO·H_2_O) + 9 H_2_O → 4CaO·Al_2_O_3_·13H_2_O + 2(3CaO·SiO_2_·3H_2_O) (9)

Al_2_O_3_·2SiO_2_+ 5(CaO·H_2_O) + 3 H_2_O → 3CaO·Al_2_O_3_·6H_2_O + 2(3CaO·SiO_2_·3H_2_O) (10)

Al_2_O_3_·2SiO_2_+ 3(CaO·H_2_O) + 6 H_2_O → 2CaO·Al_2_O_3_·SO_3_·8H_2_O+ 3CaO·SiO_2_·3H_2_O (11)

**Figure 15:**
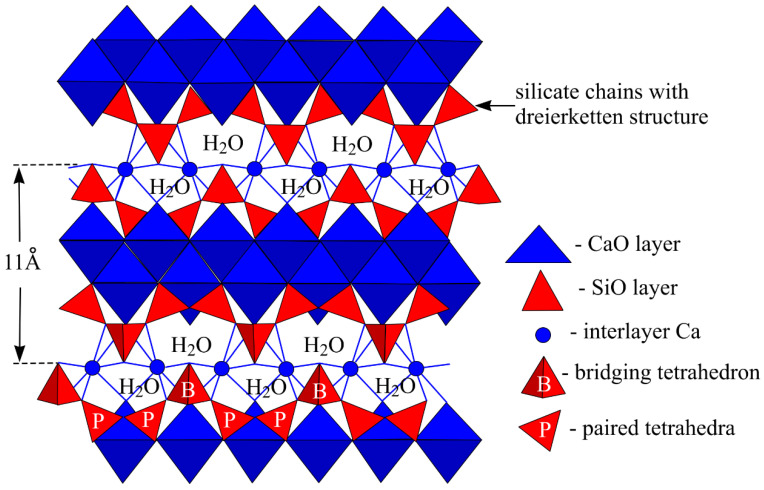
Structure model of calcium silicate hydrate.

In such cases, MKA is used for new mineral formation, not as a fine aggregate for different structure compaction. MKA in its composition has Al ions, due to which when forming tobermorite part, Si ions are replaced, forming a more resistant structure [23]. Aluminum is mainly taken up in the bridging position of the silica dreierketten structure, which increases the chain length. The aluminum uptake in C-S-H increases with the aqueous aluminum concentration [24]. A visual tobermorite structure is shown in Figure 15.

In samples C20 and C30, the cement amount was lower than in other samples. Taking into account that the main tobermorite amount depends on portlandite formed during cement hydration, then in C20 and C30 cases accordingly, this amount was lower. Previous DTG-TGA research showed that exothermic peaks were in the 920–930 °C temperature range. Ptáček [25] shows that at 925–1050 °C temperature, an amorphous SiO_2_ crystal balite separation takes place according to chemical Equation (12):

2(Al_2_O_3_·2SiO_2_) → 2Al_2_O_3_·3SiO_2_ + SiO_2_ (12)

When evaluating bending and compression results, C30 samples had the worst indicators for these properties. A conclusion can be drawn that this happened due to the small cement amount. The C20 sample bending and compression experiment results were worse than the E sample results, likely due to the fact that it also had a low cement amount in the composition. It can be concluded that the surplus of MKA, which does not take place during the tobermorite forming process when combining portlandite (CH) and is used as an aggregate in the composition, does not have a positive influence on sample mechanical properties (compression and bending). MKA takes place in the secondary tobermorite forming process as well; however, those sample amounts are not enough to change mechanical properties significantly.

During the research, the samples were hardened using hydrothermal conditions. Of course, in those conditions, quartz sand reacts with portlandite (CH), forming regular 11A tobermorite Equations (13) and (14) according to the following chemical formulas.

5(CaO·H_2_O) + 6Si_2_O→ Ca_5_Si_6_O_16_(OH)_2_·4H_2_O (13)

CaO·H_2_O+ 3CaO·Si_2_O·8H_2_O → Ca_5_Si_6_O_16_(OH)_2_·4H_2_O (14)

In the case of the MKS50 samples, MKA replaced 50% of milled quartz sand, while MKS100 sample case MKA changed the whole of quartz sand. The cement amount in these sample groups was the same when compared with E samples. DTG and TGA results did not show that MKA remained unreacted and had fulfilled its filler role. It was noted that the MKA amount in the case of MKS100 cement amount was about 26% when compared to the C20 case—25%. It can be assumed that the starting MKA reaction when forming tobermorite without using portlandite, while during hydrothermal hardening, milled quartz was more reactive than MKA, due to which during the C20 sample case there was a part of unused MKA. It can be concluded that when changing milled quartz sand, MKA was fully used for the microstructure formation, due to which there is no basis to state that it works as an aggregate.

Analyzing a dry density sample, a logical sequence can be determined that MKA lowers sample density. Mainly due to this, MKA is lighter than replaced cement or milled quartz sand. The fact that MKA lowers cement material density was determined by researcher Šeputytė-Jucikė [26]. They determined that MKA additive lowers cement rock density due to MKA additive specific surface area rise (2.5 times) when compared with Portland cement particles. Khalib [27], when researching the MKA effect on concrete materials, determined that concrete density lowers as the MKA amount rises. Significant density lowering was determined when the MKA amount went over 20%. In the C20 and C30 sample cases, density lowering when compared to the C10 samples was higher. Of course, in these cases, hydration products and their amounts, which form during hydrothermal conditions, are important. Evaluating main forming minerals, it is known that in cement systems, MKA replacing quartz forms more tobermorite while replacing cement—CSH [28]. These hydration product density is 2.18–2.6 g/cm^3^—CSH [29], while tobermorite—2.48 g/cm^3^ [30]. Due to CSH’s undefined range, it is difficult to complete a comparison; however, research results showed that it is not an essential factor that has an influence on dry density change due to its complex microstructure. This data can be used in ultrasound impulse splitting speed research result interpretation.

It is usually stated that ultrasound impulse splitting speed depends on the material density. The denser the material, the faster the speed is. Other scientists completed research showed that MKA raised tobermorite crystallinity, which is lower and more compact. Al ions that replace Si ions distribute more in a more compact way [31]. After evaluating the aforementioned MKA and cement hydration product reactions and carrying out research results, it can be stated that tobermorite crystallinity reflects ultrasound impulse splitting speed in experiment results. Samples such as C10, MKS50 and MKS100 have a significant tobermorite amount when compared to E samples, due to which ultrasound impulse splitting speed is higher in them (Figure 9). The C30 samples, as DTG research showed, have unreacted MKA, of which particle size is appropriate to fulfill a role of an aggregate. Theoretically, small MKA particles squeeze in between hydrates, densifying the macrostructure by doing so. However, practically that did not happen, and during the experiment, the microstructure formation had a stronger influence. Of course, sample pore size and amount also have significance for ultrasound impulse splitting speed, and final results correlate depending on all circumstances (hydration products, pore size and amount, density and etc.). Previous C30 sample macrostructure research showed that specific pore formation (Figure 7a) could have an influence on ultrasound impulse spreading speed. In the general case, impulse spreading speed lowered in samples after the fire effect. This happens due to hydration dehydration and extra absorbing pore formation. The highest ultrasound spreading speed changes were in E and MKS100 samples. It is related to CSH in them, which cracks in the fire effect temperature range. This fact is confirmed by TGA research as well, in which these samples have the highest mass losses in CSH cracking temperature range. The smallest ultrasound spreading speed change is in the C20 and C30 samples since these samples have the lowest hydrate formation due to lower cement amount usage in sample formation. In TGA research, these samples had the lowest mass losses in temperature ranges from 650 °C. Macrostructure research showed that sample E has a lower macro pore amount when compared to the C30 sample (Figure 6 and Figure 7). This confirms that rising MKA raises sample porosity. OM research shows that sample E formed a dense macrostructure due to fine-grained material usage in composition formation (Figure 6a); after the fire effect, cellulose fiber burns are visible (Figure 6b). OM research did not show substantial differences in macrostructure between E, MKS50, MKS100, C10 and C20 samples. Researchers [32] show that MKA amount rises the sample porosity when MKA goes over the 25% limit. Poon [33] researched MKA and determined that milled quartz sand effect that when MKA is up to 20%, it lowers capillary porosity, as well as 10% of milled quartz sand. Other scientists researched the quartz sand effect and determined that the latter’s rise in samples lowers the samples’ porosity. Schober [34] explains it as quartz sand particles squeezing into formed micropores. During the research, samples are composed of different components that, between themselves, reaction hydration and hydrothermal hardening process depend on their combinations and other circumstances. Porosity can determine forming hydrates and their amount [32]. In this case, CSH can raise the matrix’s compactness; however, CSH, which forms MKA and portlandite hydration, has an opposite effect. Absorption experiments confirmed ultrasound impulse splitting speed research results that the densest matrix structure was in MKS50 samples. Essentially, for all samples, except for the C30 samples, porosity was lower than in the E samples. In MKS50, the C10 sample cases, MKA and milled quartz sand took part in dense structure formation. MKA formed a complex Al-tobermorite, which without a structural formation, can be formed inside the micropore’s inner side. Milled quartz formed CSH, which filled nanopores. In the MKS100 sample case, the theoretically possible tobermorite amount is higher than the MKS50 or C10 sample cases, but the system does not have any milled quartz sand. However, fiber cement sample composition is complex; existing quartz sand M32, as well as can form dense structures as well as forms tobermorite on its surface. In the C20 sample cases, due to the lowered cement amount, the tobermorite amount is lower; however, the system leaves milled quartz sand, which has a lower pozzolanic effectiveness additive, which remains in the system and fills micropores.

The DTG results showed that absorbed and chemically bound water removal generally happens up to 300 °C, as well as a row of other parallel happening and overlapping processes. During the main hydrothermal hardening, a formed hydrate—11A tobermorite crystallizes into 9A tobermorite near 240 °C. Al ions containing tobermorite are slightly more resistant and hydrate near 300 °C [35]. CSH dehydration happens in the same temperature range (up to 200 °C) and forms CSH dehydration (hydrogarnet) [36]. Formed cation dehydrates. All these morphs are not separate from fixed thermal analysis methods due to their complex sample composition. At 330–340 °C temperature, an exothermic process was determined. It is determined in all samples and is related to fiber cement composition parts—cellulose fiber thermal destruction. All sample mass loss in the next stage is about 4.5% and is similar to other samples. Only insignificant exothermic effects are noted at 420–430 °C temperatures. These effects are not big and can be linked to final cellulose burnout (samples used two types of cellulose). In all samples near 573 °C temperature, an endothermic effect was determined in which polymorphic quartz change takes place. This process is not related to mass losses. From 650 °C, sample mass loss differences stand out. In these temperatures, CSH secondary dehydration starts, which is not clearly visible [37]. At higher temperatures, tobermorite hydroxyl group loss happens. In DTG research, it is determined that at 720–730 °C, when tobermorite loses its hydroxyl group and moves into a metastable state, dihydroxylation happens [38]. Near 860 °C temperature, tobermorite progresses into volostanite. This confirms the exothermic effect, which is not followed by a mass change [38]. In samples C20 and C30, near 920 °C–940 °C temperatures, a significant exothermic effect are noted when amorphous Al_2_O_3_ moves into the γ-Al_2_O_3_ form [26]. Essentially, DTG and TGA research did not show essential differences between samples, and it can be due to the complex mixture compositions needed to form samples.

Compression strength sample research results showed that MKA additive raises sample compression strength. Essentially, it is related to dense microstructure formation when Al-tobermorite and CSH have a complex effect on it as well as on its formation peculiarities. Milled quartz sand used in it also has influence, as well as quartz sand forming the framework. When compared with E samples, MKA additive replacing cement or milled quartz sand from 4.3% to 11.1% raised compression strength. The highest effect was reached when MKA was composed of 3.85% and 5% of general fiber cement material mass. Worse compression strength results of C20 and C30 were likely caused by lowered cement amount in fiber cement material. Attention is paid to the fact that C20 samples had a rather high MKA amount when compared with cement amount and theoretically did not have a strong structure. However, compression strength samples showed that these samples’ strength was only 4.9% lower than the E sample results. For comparison, the C30 results are six times worse according to percentage. It was concluded that milled quartz sand filled micropores and formed a sturdy microstructure [39,40].

Compression strength experiment results after a fire effect can be explained after evaluating sample micro and macrostructure formation as well as main composite fiber cement component and hydrate behavior in high temperatures. The C10, MKS50 and MKS100 samples in regular conditions showed the best results. The C20 samples after the fire effect had the highest compression strength loss according to percentage—30.9%. Since these sample structure formations had milled quartz sand take place by filling micropores, at high temperatures, after polymorphic quartz change, dense microstructure should have weakened, lowering the whole material’s strength by doing so.

When comparing bending strength results with compression strength results, we can see a trend. As such, we can draw a conclusion that bending strength sample results can be explained in the same way as described in compression strength sample results.

## 5. Conclusions

It was determined that when MKA additive was used to change cement fiber cement composition in autoclave samples, it raised their mechanical properties in regular conditions and after standard fire effect. A positive effect was determined in the sample where MKA did not go over 10% replacing cement. Compression strength results showed that the compression strength of these samples was 9.1% higher, while after the standard fire effect 18.58% higher than the E samples. Bending strength results showed that these samples raised bending strength by 17.02% in regular conditions and 32% after 30 min of standard fire effect when compared to the E samples. By raising the MKA amount and lowering the cement amount in samples, compression and bending strength results worsened in regular conditions and after standard fire effects.

Replacing milled quartz with MKA in 50% and 100% amounts in FCC autoclave samples raised the mechanical properties in both regular conditions and after standard fire effect. 50% of replaced quartz sand samples had the best results. Compression strength results showed that such sample compression strength was 11.05% higher after the standard fire effect and 19.67% higher than the E samples. Bending strength results showed that these samples raised bending strength in regular conditions by 14.89% and by 44% after 30 min standard fire effect when compared with E samples. Complete replacement of quartz sand did not have such effects as MKS50 samples.

Autoclave fiber cement composition sample mechanical property positive changes were determined by the MKA additive, which formed microstructure with a higher Al tobermorite amount.

## Figures and Tables

**Table 1:** Chemical composition of raw materials.

Raw Material	Oxide Content, Weight %										
	CaO	SiO_2_	Al_2_O_3_	Fe_2_O_3_	MgO	SO_3_	K_2_O	Na_2_O	TiO_2_	R_2_O	LOI
Portland cement [19]	63.23	19.25	4.96	3.40	2.91	2.74	0.88	0.12	-	-	1.32
Quartz sand [20]	0.72	97.89	0.60	0.46	-	0.06	-	-	-	0.10	0.17
MKA [21]	0.10	51.80	34.20	0.50	0.1	-	-	0.60	0.60	-	11.5

**Table 2:** The number of raw materials in the different mixtures in percentages.

Mixture Markings	Sand M32	Sand M300	Portland Cement	Cellulose Fiber	Aluminum Hydroxide	Kaolin	MKA
E	41.05	10	38.04	7.09	2.06	1.76	-
C10	41.05	10	34.24	7.09	2.06	1.76	3.8
C20	41.05	10	30.44	7.09	2.06	1.76	7.6
C30	41.05	10	26.64	7.09	2.06	1.76	11.4
MKS50	41.05	5	38.04	7.09	2.06	1.76	5
MKS100	41.05	-	38.04	7.09	2.06	1.76	10
